# A Rare Case Report of COVID-19 and Leptospirosis Co-infection Triggering Acute Myocarditis

**DOI:** 10.7759/cureus.92948

**Published:** 2025-09-22

**Authors:** Ismail Mohammed, Arslan Baig, Ashitha P Kaniyam Parambil, Onesi Stephen Ogedengbe

**Affiliations:** 1 Internal Medicine, Manchester Royal Infirmary Manchester Foundation Trust, Manchester, GBR; 2 Geriatrics, Manchester Royal Infirmary Manchester Foundation Trust, Manchester, GBR; 3 Respiratory Medicine, Manchester Royal Infirmary Manchester Foundation Trust, Manchester, GBR; 4 Acute Internal Medicine, Manchester Royal Infirmary Manchester Foundation Trust, Manchester, GBR

**Keywords:** covid 19 and leptospirosis, leptospirosis, leptospirosis complications, leptospirosis with cardiovascular complications, leptospirosis with severe clinical manifestation, liver dysfunction with leptospirosis

## Abstract

Leptospirosis is a globally prevalent zoonotic infection, more common in tropical climates but increasingly recognised in temperate regions, such as the United Kingdom. Its clinical presentation varies widely, ranging from mild flu-like symptoms to severe complications including pulmonary haemorrhage and multi-organ failure, making diagnosis challenging. We report the case of a 25-year-old male who presented with a three-day history of fever, myalgia, non-productive cough, and shortness of breath. He tested positive for SARS-CoV-2 and was diagnosed with COVID-19 pneumonitis. His condition deteriorated, necessitating ICU admission and mechanical ventilation. During his ICU admission, he suffered complications such as pulmonary haemorrhage, a known complication of leptospirosis. Despite initial management with corticosteroids and broad-spectrum antibiotics, his liver function worsened, and echocardiography showed reduced ejection fraction with elevated troponin, suggesting myocarditis. A leptospirosis screen was sent and returned positive on day 14. Retrospective history revealed exposure to rats in his residence. The patient was started on oral doxycycline, which led to steady clinical improvement and eventual discharge. This case highlights the diagnostic difficulty in distinguishing leptospirosis from COVID-19 due to overlapping features. Early signs-including deranged liver function, pulmonary haemorrhage, and cardiac involvement-were present but not initially attributed to leptospirosis. Delayed consideration of zoonotic exposure contributed to a delay in targeted therapy. Clinicians should maintain a high index of suspicion for leptospirosis in patients presenting with multi-organ involvement, particularly in the setting of potential environmental exposure. Early diagnosis and initiation of appropriate antimicrobial therapy are vital to improving patient outcomes.

## Introduction

Leptospirosis is an important spirochaetal infection caused by a bacterium of the genus Leptospira. It is a zoonotic infection that is commonly found in tropical regions of the world but not limited to these endemic regions. The mortality rate due to leptospirosis infection remains significant due to lack of infrastructure or clinical suspicion [1]. It is also known that when not treated or diagnosis is delayed, leptospirosis can cause multi-organ failure involving major organs, such as the kidneys, lungs and liver [2]. In non-endemic areas, it is very easy to miss the diagnosis of leptospirosis due to low index of clinical suspicion [3]. According to the animal-associated infections annual report in the UK, there were 52, 70 and 102 cases of leptospiral infections identified in the years 2022, 2023 and 2024, respectively. The number of cases each year has been increasing steadily [4]. Interestingly, the recent COVID-19 pandemic, which caused devastation around the world, bumped COVID-19 as a top differential when patients arrive with respiratory symptoms. However, some cases have been reported where leptospirosis infection has presented mimicking a COVID-19 presentation, subsequently making diagnosis difficult [5]. Additionally, there seems to be a general lack of awareness or education around leptospirosis infection, even though it is one of the most common zoonotic infections and with development of easier air travel, even non-endemic areas may see a rise in the number of patients requiring hospital admissions. 

## Case presentation

A 25-year-old gentleman with no significant past medical history was brought to the emergency department due to a three-day history of fever, shortness of breath, non-productive cough, and generalised myalgias, primarily affecting the chest, lower back, and legs. He was a non-smoker, non alcoholic, and had no illicit drug use, and had no recent travel outside the UK. He worked at a UK-based supermarket.

Examination and initial assessment

**Figure 1:**
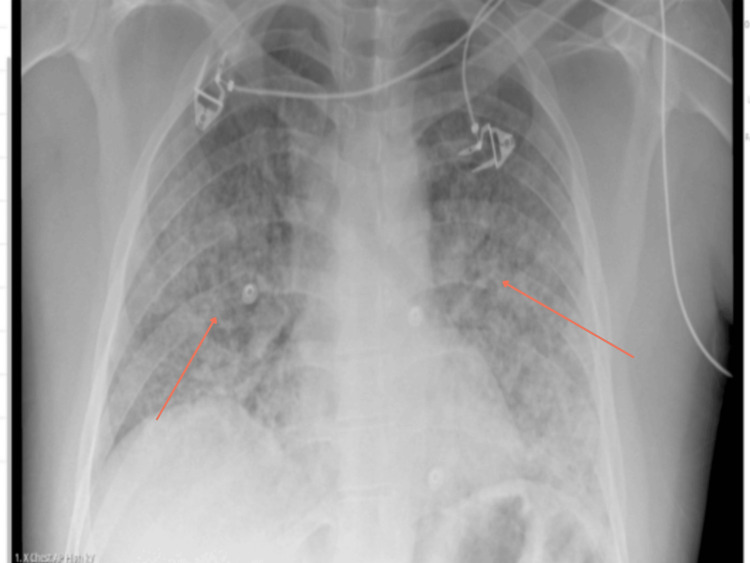
Initial Chest X-ray showing patchy opacification bilaterally suggestive of infection

**Figure 2:**
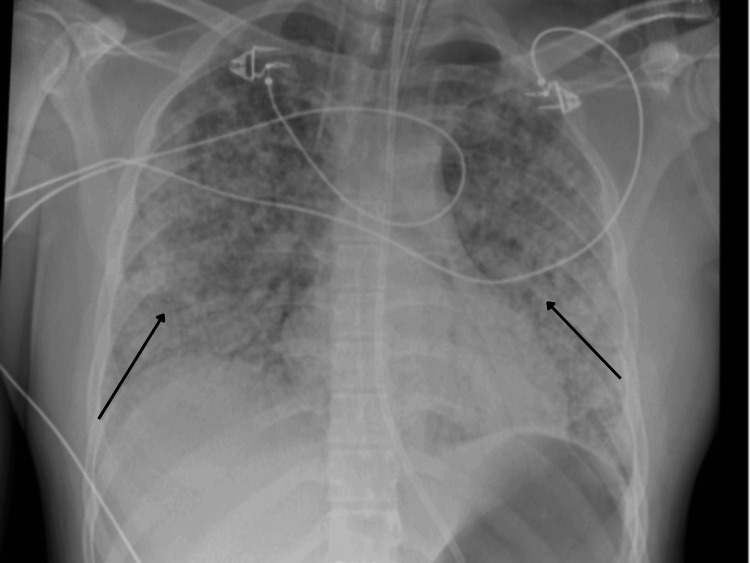
Repeat Chest X-ray four hours from initial Chest X-ray showed worsening bilateral infiltrates

**Figure 3:**
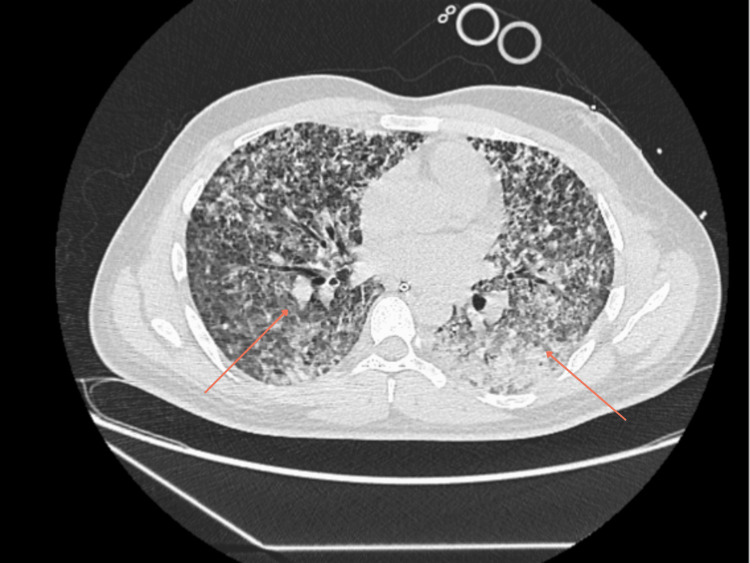
High Resolution CT Thorax Demonstrates extensive bilateral dense airspace shadowing, which is worse in the lower lobes, especially on the left. No pulmonary haemorrhage seen.

On presentation, the patient was febrile, hypotensive, hypoxic and had tachycardia. Physical examination revealed reduced air entry on the right side of the chest. His observations and initial investigations are as shown in Table 1 and Table 2. Furthermore, initial chest radiographs showed patchy opacification. A repeat chest radiograph was done four hours apart to confirm position of the nasogastric (NG) tube and central venous catheter (CVC) positions and incidentally showed worsening of bilateral pulmonary infiltrates, as demonstrated in Figure 1 and Figure 2, respectively. Subsequently, a high-resolution CT-Thorax demonstrated extensive bilateral air base shadowing, which was worse in the bases of the lungs, but no evidence of pulmonary haemorrhage was reported, as seen in Figure 3.    

**Table 1:** Vital Signs

Vital sign	Result
Heart rate	144 bpm
Respiratory rate	22 breaths/min
Blood pressure	88/56 mmHg
Temperature	38.4°C
SpO₂	96% on 15 L/min oxygen via non-rebreather mask

**Table 2:** Initial Investigations ALP: alkaline phosphatase, ALT: alanine aminotransferase, GFR: glomerular filtration rate, NT-proBNP: N-terminal pro-brain natriuretic peptide, PCR: polymerase chain reaction

Test			Result		Reference range
White cell count (WCC)			13.1 ×10⁹/L		4.0 – 11.0 ×10⁹/L
Neutrophils			12.19 ×10⁹/L		1.80 – 7.50 ×10⁹/L
Haemoglobin (Hb)			129 g/L		130 – 180 g/L
Platelet count			33 ×10⁹/L		150 – 400 ×10⁹/L
C-reactive protein (CRP)			232 mg/L		1-5 mg/L
Procalcitonin			17.00 ug/L		< 0.5 µg/L
Urea			15.9 mmol/L		2.5 – 7.8 mmol/L
Creatinine			260 µmol/L		59 – 104 µmol/L
Estimated GFR			28 mL/min/1.73m²		≥ 90 ml/min/1.73m²
ALT			82 IU/L		1-50 IU/L
ALP			201 U/L		30 – 130 U/L
Bilirubin (Total)			62 µmol/L		0 – 21 µmol/L
NT-proBNP			3740 ng/L		0-400 ng/L
Troponin T			677 ng/L		0-14 ng/L
Arterial blood gas			Type 1 respiratory failure		
SARS-CoV-2			Positive by PCR		

Initial treatment on admission 

In the Accident and Emergency Department (A&E), he was treated for acute respiratory distress syndrome secondary to chest infection and was commenced on high flow oxygen, IV fluids and IV antibiotics. A Chest X-ray (Figure 1) revealed bilateral patchy opacification throughout lungs. His respiratory viral swabs results were positive for SARS-CoV-2 RNA polymerase chain reaction (PCR)-positive infection. Considering worsening respiratory distress, he was then reviewed by the Critical Care team. 

ICU course 

**Figure 4:**
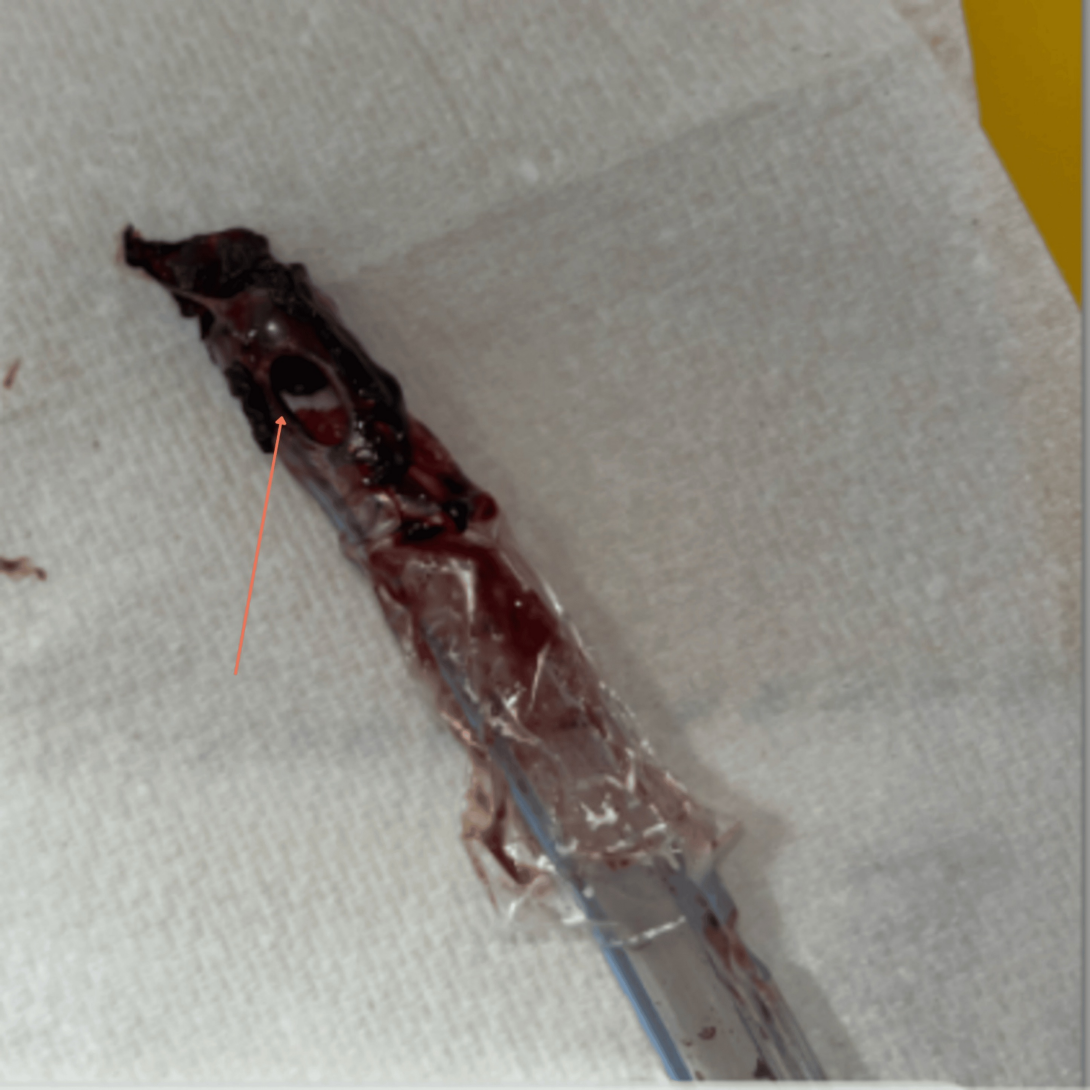
Thick blood clot at the tip of endotracheal tube due to pulmonary haemorrhage

The patient was transferred to ICU following rapid clinical deterioration and was intubated and mechanically ventilated. A provisional diagnosis of acute respiratory distress syndrome (ARDS) secondary to COVID-19 pneumonia was made. Therefore, IV co-amoxiclav was escalated to piperacillin-tazobactam and IV clarithromycin; additionally IV dexamethasone was also added to his treatment plan in view of worsening pulmonary symptoms. Furthermore, linezolid was added on to cover for worsening pneumonia as advised by the Infectious Disease team; however was stopped after two days due to worsening thrombocytopenia, which is a known complication of leptospirosis. Furthermore, bronchoscopy revealed secretions, suggestive of pulmonary haemorrhage, with a thick blood clot observed at the endotracheal tube tip, shown in Figure 4. 

Along with ARDS, myocarditis was also suspected due to elevated troponin levels and echocardiography showing left ventricular ejection fraction (LVEF) 45-50% with grade I diastolic dysfunction. Hence, he was started on a beta-blocker and an angiotensin-converting enzyme (ACE) inhibitor. Despite these therapeutic interventions, his liver function progressively worsened. Considering that his non-invasive liver screen was negative and normal imaging, there were concerns for drug-induced liver injury or ischemic hepatitis. Subsequently, as other non-invasive liver screens were normal, a potential leptospiral infection was suspected by the gastroenterologist, who advised completing a leptospirosis IgM (enzyme immunoassay (EIA)) screen in order to explain symptoms and deranged liver function.

Interim clinical course

**Figure 5:**
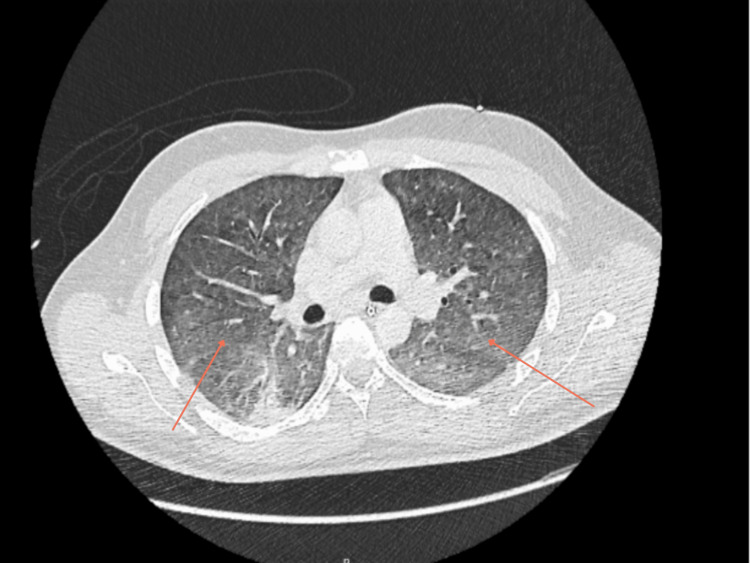
CT Thorax Abdomen Pelvis on day six Shows interval improvement in pulmonary opacities, however extensive bilateral diffuse ground glass component

IV piperacillin-tazobactam was switched to IV meropenem in addition to the previously mentioned antibiotics in view of rising white cell counts. The case was discussed at a multidisciplinary lung team (MDT) meeting. Endo-bronchial ultrasound-guided lymph node biopsy was recommended. The follow-up blood tests and microbiology and serology at this stage are shown in Table 3 and Table 4, respectively. A CT thorax was done at this stage to check for resolution, which showed interval improvement of opacities; however, it also showed bilateral diffuse ground-glass component, as shown in Figure 5. 

**Table 3:** Follow up blood tests ALP: alkaline phosphatase, ALT: alanine aminotransferase, GFR: glomerular filtration rate

Test	Results					Reference range
	Day 6	Day 14	Day 20 (Discharge Day)	10 Days after Discharge	3 Weeks after Discharge	
White cell count (WCC)	44.9×10⁹/L	6.2×10⁹/L	5.8 ×10⁹/L	7.5 ×10⁹/L	8.8 ×10⁹/L	4.0–11.0 ×10⁹/L
Neutrophils	34.80 ×10⁹/L	4.13×10⁹/L	2.97×10⁹/L	3.65×10⁹/L	4.46×10⁹/L	1.8 – 7.5 ×10⁹/L
Haemoglobin (Hb)	93.0 g/L	116 g/L	113 g/L	126 g/L	132 g/L	130 – 180 g/L
Platelet count	364.0 ×10⁹/L	489×10⁹/L	348×10⁹/L	436×10⁹/L	376×10⁹/L	150 – 400 ×10⁹/L
C-reactive protein	23 mg/L	3 mg/L	<1 mg/L	<1 mg/L	<1 mg/L	0-5 mg/L
Procalcitonin	0.93 µg/L					< 0.5 µg/L
Urea	8.4 mmol/L	10.3mmol/L	4.7mmol/L	4.2mmol/L	5.6mmol/L	2.5 – 7.8 mmol/L
Creatinine	56 µmol/L	65 µmol/L	52µmol/L	68µmol/L	61µmol/L	59 – 104 µmol/L
Estimated GFR	>90 ml/min	>90 ml/min/1.73m²	>90 ml/min/1.73m²	>90 ml/min/1.73m²	>90 ml/min/1.73m²	≥ 90 ml/min/1.73m²
ALT	149 IU/L	393 IU/L	227 IU/L	140 IU/L	64 IU/L	1-50 IU/L
ALP	126 U/L	147 U/L	127 U/L	103 U/L	81 U/L	30 – 130 U/L
Bilirubin (Total)	173 µmol/L	140umol/L	66 umol/L	29 umol/L	18 umol/L	0-21 umol/L

**Table 4:** Microbiology and Serology PCR: polymerase chain reaction, BAL: bronchoalveolar lavage, EIA: enzyme immunoassay

Test	Result
Aerobic and non-aerobic blood cultures	No growth
Mycoplasma pneumonia PCR	Negative
Legionella urinary antigen	Negative
Pneumococcal urinary antigen	Negative
Acid fast bacilli stain	Negative
Tuberculosis culture	No growth
Malaria enzyme-linked immunosorbent assay (ELISA)	Negative
HIV 1+2 antibody and p24 antigen	Negative
Legionella BAL culture	Negative
Aspergillus galactomannan antigen	Negative
Fungal beta D-glucan	Negative
Leptospira IgM (EIA)	Positive

Further outcome

The patient was extubated after one week and subsequently stepped down to the ward. Intravenous meropenem was administered from day five until day 11, during which he showed progressive clinical improvement and normalisation of inflammatory markers. Despite this, his liver function continued to deteriorate, with worsening jaundice. Bilirubin levels began to improve from day 11, followed by improvement in alanine transaminase (ALT) from day 14. On day 14, Leptospira IgM (EIA) returned positive, and oral doxycycline was initiated for seven days. Following initiation of targeted therapy, liver function continued to improve steadily, and the patient was discharged in a stable condition.  

## Discussion

Leptospirosis is one of the most widespread zoonotic infections globally, with a higher incidence in tropical regions and areas experiencing heavy rainfall [6]. However, it has also been reported in temperate countries such as the United Kingdom [7,8]. The clinical presentation of leptospirosis is highly variable, ranging from mild flu-like symptoms to severe manifestations, such as pulmonary haemorrhage and multi-organ failure [9]. Its varied and multisystem involvement often hinders timely diagnosis, especially in non-endemic areas, where it is not commonly considered as a differential.  

Transmission occurs primarily through direct contact with the urine or faeces of infected animals, or indirectly through contaminated water or soil [10]. Furthermore, cases have been reported following recreational exposure to contaminated water bodies [11]. The diagnostic challenge intensifies when leptospirosis mimics other common infections. For instance, during the COVID-19 pandemic, several cases of leptospirosis with pulmonary involvement were misdiagnosed as COVID-19 pneumonitis due to overlapping clinical and radiological findings [12].  

In this case, a 25-year-old male presented to the emergency department with a three-day history of fever, shortness of breath, non-productive cough, and generalized myalgia. His initial workup was suggestive of sepsis, with elevated white cell count, inflammatory markers, and a positive SARS-CoV-2 PCR. Chest radiographs showed diffuse patchy opacities that rapidly progressed within hours, further supporting a working diagnosis of COVID-19 pneumonitis. Consequently, the patient was admitted to the ICU, intubated, and treated with corticosteroids and broad-spectrum antibiotics.  

A notable finding during bronchoscopy was the presence of a thick endotracheal clot indicating pulmonary haemorrhage - an established complication of pulmonary leptospirosis [13]. Additionally, the patient demonstrated elevated troponin levels and echocardiographic evidence of mildly reduced LVEF (45-50%) with grade I diastolic dysfunction, raising suspicion for myocarditis. ACE inhibitors and beta blockers were initiated accordingly.  

Despite appropriate management for presumed COVID-19 pneumonitis and suspected bacterial superinfection, the patient’s liver function progressively deteriorated. The pattern of deranged liver function tests (LFTs) prompted further evaluation, including a leptospirosis screen. While the literature presents mixed findings on hepatic involvement in leptospirosis, recent studies suggest that mild to moderate elevations in liver enzymes and bilirubin may occur [14]. In this patient, the early abnormal LFTs could have been attributed to leptospirosis rather than COVID-19 or myocarditis alone.  

A detailed exposure history was only obtained retrospectively, revealing that the patient had experienced a rodent infestation in his residence. This is significant, as rodents are a primary reservoir for Leptospira species, and multiple cases of leptospirosis following rat exposure have been documented [15]. Unfortunately, a lack of early inquiry into occupational and environmental exposures delayed the diagnosis.  

Coinfection with SARS-CoV-2 may have influenced the clinical trajectory and masked features of leptospirosis, particularly pulmonary involvement, which is well-documented in severe cases [16]. Although the patient showed pulmonary improvement and was successfully extubated, liver dysfunction persisted. On hospital day 14, leptospirosis serology returned positive, and the patient was commenced on oral doxycycline, the first-line therapy for leptospirosis [17]. Following antibiotic initiation, his liver function improved steadily, and he was discharged in stable condition.  

This case underscores several missed diagnostic opportunities: Firstly, a lack of early environmental and occupational history, which could have raised suspicion for leptospirosis. Additionally, deranged LFTs in an otherwise healthy young male should have prompted consideration of alternative diagnoses. Presence of of endotracheal tube clotting revealed during bronchoscopy was suggestive of pulmonary haemorrhage, which is a well-known complication of leptospirosis.

## Conclusions

Leptospirosis remains a clinically important but often under-recognised zoonotic disease. In this case, co-infection with COVID-19 led to diagnostic overshadowing, delaying appropriate treatment. Severe leptospirosis infection can lead to Weil's disease, which is strongly associated with serious lung complications, including ARDS and pulmonary haemorrhage, which could have been attributed to leptospirosis rather than a COVID-19 infection.

Clinicians, particularly in non-endemic regions, must remain vigilant for atypical presentations and consider zoonotic exposures as part of routine history-taking. Early recognition and targeted antibiotic therapy are crucial, as delays in diagnosis can result in significant morbidity or mortality. This report aims to enhance awareness of leptospirosis, encourage its inclusion in differential diagnoses, and emphasise the importance of thorough environmental history in patients with undifferentiated febrile illnesses.
